# Black Soldier Fly Larvae Can Effectively Degrade Oxytetracycline Bacterial Residue by Means of the Gut Bacterial Community

**DOI:** 10.3389/fmicb.2021.663972

**Published:** 2021-06-15

**Authors:** Cuncheng Liu, Huaiying Yao, Cunwen Wang

**Affiliations:** ^1^Research Center for Environmental Ecology and Engineering, School of Environmental Ecology and Biological Engineering, Wuhan Institute of Technology, Wuhan, China; ^2^Key Laboratory of Green Chemical Process of Ministry of Education, Key Laboratory of Novel Reactor and Green Chemical Technology of Hubei Province, School of Chemical Engineering and Pharmacy, Wuhan Institute of Technology, Wuhan, China; ^3^Zhejiang Key Laboratory of Urban Environmental Processes and Pollution Control, Ningbo Urban Environment Observation and Research Station, Chinese Academy of Sciences, Ningbo, China; ^4^Key Laboratory of Urban Environment and Health, Institute of Urban Environment, Chinese Academy of Sciences, Xiamen, China

**Keywords:** oxytetracycline bacterial residue, *Hermetia illucens*, bioremediation, antibiotic resistance genes, microbiota

## Abstract

Antibiotic bacterial residue is a unique hazardous waste, and its safe and effective disposal has always been a concern of pharmaceutical enterprises. This report presents the effective treatment of hazardous waste—antibiotic bacterial residue—by black soldier fly larvae (larvae), oxytetracycline bacterial residue (OBR), and soya meal with mass ratios of 0:1 (soya), 1:20 (OBRlow), and 1:2 (OBRhigh), which were used as substrates for larval bioconversion. Degradation of OBR and oxytetracycline, the bacterial community, the incidence of antibiotic resistance genes (ARGs) and the bacterial function in the gut were examined. When the larvae were harvested, 70.8, 59.3, and 54.5% of the substrates had been consumed for soya, OBRlow and OBRhigh; 65.9 and 63.3% of the oxytetracycline was degraded effectively in OBRlow and OBRhigh, respectively. The larval bacterial communities were affected by OBR, abundant and various ARGs were discovered in the gut, and metabolism was the major predicted function of the gut. These findings show that OBR can be digested and converted by larvae with gut bacteria, and the larvae can be used as a bioremediation tool for the treatment of hazardous waste. Finally, the abundant ARGs in the gut deserve further attention and consideration in environmental health risk assessments.

## Introduction

During the production of antibiotics, waste antibiotic bacterial residue is generated. Originally, antibiotic bacterial residue was used for feed addition, as it is rich in protein, fat, cellulose, and other components (Guo et al., 2012). However, numerous toxic compounds, such as antibiotic residues, heavy metals, fermentation byproducts, metabolic products, and byproducts, remain in these bacterial residues (Guo et al., 2012). Antibiotic bacterial residue is a unique hazardous waste, and its safe and effective disposal has always been a concern of pharmaceutical enterprises. In China, more than 248,000 tons of antibiotics are produced, and 2 million tons of antibiotic bacterial residue are generated every year. Because of its broad activity against infection, oxytetracycline is produced and used in human and veterinary medicine in large quantities, resulting in the generation of a large amount of oxytetracycline bacterial residue (OBR). OBR is a fermentative residue from *Streptomyces rimosus* and inevitably contains high concentrations of oxytetracycline due to incomplete extraction (Gong et al., 2020). This fermentative residue contains not only dense nutrients but also some toxic and harmful substances, such as metabolic byproducts (Guo et al., 2012). In particular, oxytetracycline residues may lead to the spread of bacterial resistance and ARGs to the environment. Therefore, considering the potential ecotoxicological risks, OBR has been regarded as a typical hazardous waste (Meng et al., 2013; Wang B. et al., 2020; Wang M. et al., 2020), and safe and effective treatment methods for OBR are urgently needed because of increasing pressure from environmental issues in recent years. Safe, practical, and effective methods for dealing with antibiotic bacterial residue include composting, anaerobic digestion, combination with yeast extract products, mixing with coal water slurry, and modification of the bacterial residue to form activated carbon (Kakimoto and Funamizu, 2007; Kakimoto et al., 2007; Zhou et al., 2011, 2012; Li et al., 2012). However, these methods have high processing costs and produce varying degrees of secondary pollutants.

Black soldier fly (*Hermetia illucens*) larvae can not only effectively digest organic waste but also decompose toxic bacteria during the conversion of organic wastes, which reduces the accumulation of wastes and their harm to the environment (Liu et al., 2019). Because of their biological characteristics and wide feeding range, larvae have been extensively used in the field of organic waste disposal (Liu et al., 2021), which is beneficial to the recycling of wastes and environmental protection (Liu et al., 2019). In bioconversion, the proteins and lipids in organic waste are converted into polypeptides (Park et al., 2015). Great progress has been made in evaluations of the nutritional value of *Hermetia illucens*, which has good development prospects. For example, the protein in larvae is used as animal feed (Schiavone et al., 2017) or formula feed for raising chickens, pigs, and fish; lipids are used for the production of biodiesel as an energy material (Wang et al., 2017); and the larval body can be used for the extraction of antimicrobial peptides (Vogel et al., 2017), chitin, and chitosan (Abdou et al., 2008). As previously reported, microbial metabolic capacity is the key to the reduction and recycling of organic waste (Yu et al., 2007), and the composition of larval intestinal microorganisms (fungi and bacteria) (De Smet et al., 2018; Bonelli et al., 2019; Bruno et al., 2019) and enzymes (Kim et al., 2011) is complex. Therefore, the larval intestinal microbial ecosystem may be the key and play an important role during the bioconversion of organic wastes by larvae (Zheng et al., 2012; Bertinetti et al., 2019; Wynants et al., 2019). These synergistic microorganisms in the larval gut enable more efficient conversion of organic waste (Abduh et al., 2017). Interestingly, larvae can also digest rice straw (Liu et al., 2021) and degrade mycotoxins, pesticides, and antibiotics (Bosch et al., 2017; Purschke et al., 2017; Liu et al., 2020), which effectively reduces the transmission and migration of these organic pollutants in environmental media (Lalander et al., 2016; Cifuentes et al., 2020). In addition, based on the performance of bacterial communities and the incidence of ARGs in the larval gut, oxytetracycline can be degraded effectively by larvae (Liu et al., 2020). However, it remains challenging to ensure larval reliability and high conversion performance for degrading oxytetracycline with different substrates (Gold et al., 2020). Therefore, it is very important to understand the ability of larvae to digest the substates mixed with OBR and to degrade the oxytetracycline remnants in the OBR.

In detail, the degradation efficiency of oxytetracycline remaining in the OBR by larvae was investigated, and the key roles and functional potential of the larval bacterial community were evaluated. In this work, OBR was selected as the substrate for larval treatment. The conversion and disposal processes of OBR by larvae were analyzed, and the degradation processes of oxytetracycline remaining in the OBR degraded by the larvae were examined. To explain the bacterial ecological mechanism of the degradation of oxytetracycline and bioconversion of OBR by larvae, changes in the gut bacterial community structure, composition, the key roles and functional potential, and the incidence of ARGs and mobile genetic elements (MGEs) in the larval gut were investigated. This study will help improve our knowledge regarding larval performance to dispose of hazardous waste—antibiotic bacterial residue.

## Materials and Methods

### Oxytetracycline Bacterial Residue (OBR)

OBR, which contained 32.1% crude protein (dry weight) and 11.2% crude fat (dry weight), was obtained from an oxytetracycline pharmaceutical factory in Hebei Province, China. The OBR was dried at 105°C, crushed with a mechanical crusher and passed through a 250-μm sieve. A diagram of dried OBR is shown in Supplementary Figure 1. Initially, different antibiotics (oxytetracycline, aureomycin, doxycycline, ofloxacin, enrofloxacin, sulfadiazine, sulfamethoxine, sulfadimidine) were detected as target antibiotics in OBR, and only oxytetracycline antibiotics were detected at a concentration of 9126.88 mg kg^–1^ (dry weight). Dried and crushed OBR was used as the diet for the larvae.

### Procurement and Breeding of Larvae

To illustrate the ability of the larvae themselves to degrade antibiotics, eggs were obtained from the breeding base for larvae of the Resources Utilization and New Energy Development team at the Wuhan Institute of Technology. Similarly, a mixed substrate of wheat bran (20 wt%), corn flour (20 wt%), and chick starter (60 wt%) with a water content of 60–70% was used to activate eggs in a biochemical incubator with a constant temperature of 29°C and humidity of 65% for 3 days (Wang et al., 2017; Feng et al., 2018; Liu et al., 2020). Wheat bran, corn flour, chick starter and soya meal were purchased from the Wuhan farmers’ market.

Because the soya meal was rich in protein, which was helpful for larval growth and development, the substrate for rearing larvae was a uniform mixture of OBR and soya meal. In a preliminary experiment, we found that larvae did not grow when the content of OBR was 100 and 50% in the substrates uniformly mixed with soya meal (mass ratio, dry weight). Because the concentration of oxytetracycline in the OBR was as high as 9126.88 mg kg^–1^ (dry weight), which exceeded the tolerance of the larvae, the larvae could not directly degrade the bacterial residue. Thus, the effective utilization of OBR by black soldier fly larvae might require a combination with other organic matter that could be converted and degraded by larvae. Therefore, the degradation of 0:1, 1:20, and 1:2 (OBR:soya meal) was analyzed. Here, the treatments containing only soybean meal, a low amount of OBR, and high amount of OBR were defined as soya, OBRlow and OBRhigh, respectively.

The feed mixture was formulated as 0:1 (soya), 1:20 (OBRlow), and 1:2 (OBRhigh) OBR:soya meal (mass ratio, dry weight) with OBR percentages of 0% (wt%), 4.8% (wt%), and 33.3% (wt%) and initial oxytetracycline concentrations of 0, 434.4, and 3042.3 mg kg^–1^, respectively. In this experiment, 3-day-old larvae with an initial fresh weight of 33.6 mg (10 larvae) ^–1^ were selected for degradation. Then, a breeding box (25 × 15 × 10 cm) that contained 250 g of well-mixed substrates (dry weight) and a biochemical incubator that could maintain a constant temperature (29°C) and humidity (65%) were used for larval reproduction and cultivation with three replicates; larvae were selected randomly for the determination of average weight. Initially, 10 g with approximately 3,050 ± 50 larvae were introduced in one breeding box, resulting in a total of nine tests. Previously, the weight of the breeding box was known, and then the degradation efficiency of the substrate could be estimated based on the changes in larval weight. The specific details and conditions followed a previous report (Liu et al., 2020).

### Oxytetracycline in the OBR

The degradation of oxytetracycline and bioconversion of OBR were investigated in batch systems over a period of 7 days of cultivation before more than 60% of the larvae turned prepupal and ceased eating (Holmes et al., 2013; Liu et al., 2020). During cultivation, a certain amount of homogenized substrate (approximately 2 g) was collected for oxytetracycline extraction and determination at the same time every day. Before the larvae began to pupate, they migrated and were harvested from the substrate. Then, the larval guts were emptied for 24 h to determine the accumulation of oxytetracycline in the larval bodies. The oxytetracycline in the substrate and larval bodies was extracted and determined as previously described (Liu et al., 2020). Briefly, the substrate and larval bodies were first freeze-dried, crushed, and screened with sieves; then, the oxytetracycline in 1 g of each sample was extracted with a total of 15 mL Na_2_EDTA:McIlvaine:methanol (1:1:2 vol ratio) buffer solution with the assistance of vortices and ultrasonication. The extracts were successively loaded and eluted for purification using Oasis HLB cartridges (Agela, 500 mg, 6 mL); finally, the concentrations of oxytetracycline in the reconstituted solution were measured by HPLC (high-performance liquid chromatography, Agilent Technologies 1260 Infinity, United States).

### DNA Extraction and Analysis of the Larval Gut Bacterial Community

Ten larvae were selected randomly for aseptic treatment of the larval body and DNA extraction from the larval gut. Chloroform was used to kill the larvae immediately and prevent the excretion of gut, 2% sodium hypochlorite (NaOCl) solution was used for sterilization of the larval body surface for 10 s, and a total of 50 mL of sterilized water was used to clean the larval body surface five times. After aseptic treatment and dissection, the dissected guts were collected for DNA extraction using a FastDNA Spin Kit for feces (MP Biomedicals, Illkirch, France), and then 1.0% agarose gel electrophoresis and spectrophotometric analysis (NanoDrop ND-1000, Thermo Fisher Scientific) were used to check the concentration and quality of the gut DNA. The extracted DNA was amplified using the bacterial 16S rRNA gene with the universal primers 314F and 806R, the premixed samples were sent for sequencing on the Illumina MiSeq platform, and then the high-throughput sequencing data for the gut DNA were analyzed by Quantitative Insights Into Microbial Ecology (QIIME). The total bacterial abundance of larval gut DNA was estimated by real-time quantitative PCR (RT-qPCR) with the universal primers 515F and 907R and quantified by a standard curve. The abundance and diversity of ARGs in the larval gut were investigated by high-throughput quantitative PCR (HT-qPCR) with 296 primer sets targeting 285 ARGs, 10 MGEs [8 transposases, one class 1 integron-integrase gene (intI1), and one clinical class 1 integron-integrase gene (cintI1)], and 1 16S rRNA marker gene (Zhu et al., 2018). The specific methods, details, and procedures involved in the above experiments were as previously described (Liu et al., 2020). In addition, PICRUSt (phylogenetic investigation of communities by reconstruction of unobserved states) was used to directly identify larval gut bacterial function with 16S rRNA information (Langille et al., 2013; Liu et al., 2021).

### Statistical Analysis

In this article, all data are expressed as the mean values ± standard error and are presented in figures with triplicate samples. The consumption of diet, the concentration of oxytetracycline in the diet at any time, the degradation efficiency, and the residual oxytetracycline were calculated with Supplementary Equations 1–4. The bioinformatics analysis of gut bacterial community microorganisms (diversity, composition, etc.) was analyzed using R 3.3.1 with vegan 2.4-3. Differences among samples were analyzed using single-factor analysis of variance (ANOVA) and principal coordinate analysis (PCoA) based on the weighted UniFrac distance. Significant differences were detected at the 0.05 level. The abundance and diversity of ARGs and MGEs were analyzed by SmartChip HT-qPCR (WaferGen Inc., Fremont, CA, United States) software with a cycle threshold (CT) of 31 and an amplification efficiency of 0.9–1.1, as previously described (Zhu et al., 2018; Li et al., 2019; Liu et al., 2020). Microsoft Excel and IBM SPSS Statistics version 25 were used for the data processing *t*-tests. The bar charts, scatter diagrams, pie charts, and heatmaps were implemented with OriginPro.

## Results

### Degradation of Oxytetracycline and OBR

**FIGURE 1:**
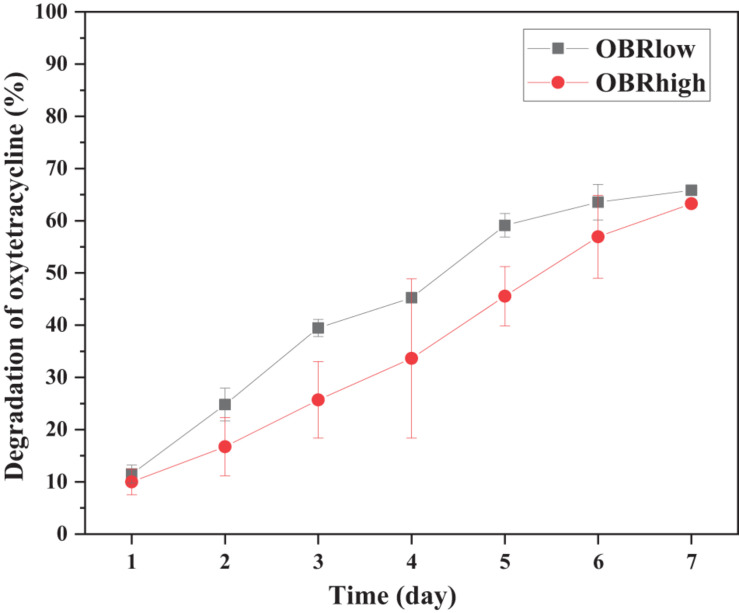
Efficiency of oxytetracycline degradation by larvae. Each point is the average of triplicate samples (mean ± SE). OBRlow and OBRhigh represent the treatments containing low amounts of OBR (OBR:soya, 1:20) and high amounts of OBR (OBR:soya, 1:2), respectively.

The substrates with and without OBR were both degraded by larvae (Supplementary Figure 2A). As the content of OBR increased, the consumption of substrate decreased; the consumption of soya, OBRlow, and OBRhigh decreased by 70.8, 59.3, and 54.5%, respectively, and 65.9 and 63.3% of the oxytetracycline in the OBR of OBRlow and OBRhigh, respectively, was effectively degraded (Figure 1). From the substrate degradation, the larvae significantly (ANOVA test, *P* < 0.001) gained weight: from an initial weight of 33.6 mg 10 larvae^–1^ (fresh weight) to a final weight of approximately 555.6–843.0 mg 10^–1^ larvae (fresh weight) after harvest (Supplementary Figure 2B and Supplementary Table 1). When the OBR content increased, the larval weight decreased, and larval development was significantly affected by the different substrates (ANOVA test) in proportion to substrate consumption. Importantly, oxytetracycline was not detected in the larval tissue, even though the larvae digested the substrates that contained OBR.

### Larval Bacterial Community

#### Abundance and Diversity of the Larval Bacterial Community

**FIGURE 2:**
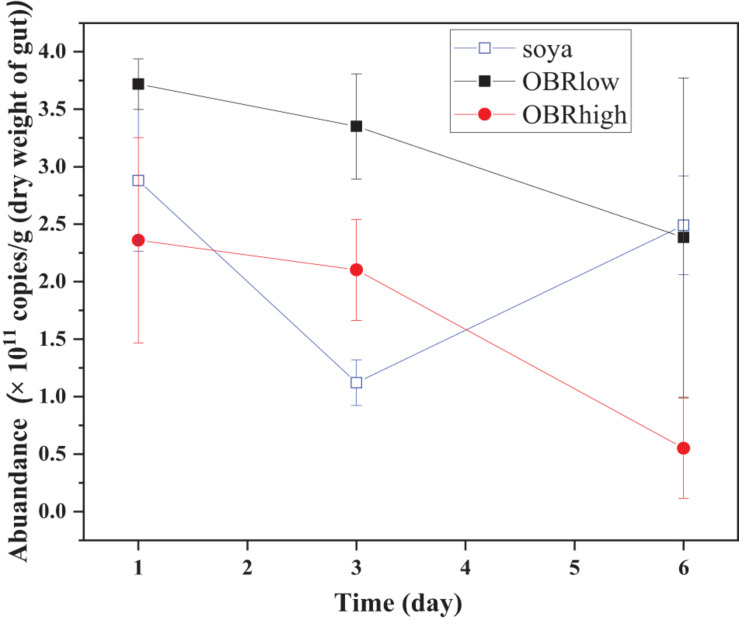
Real-time quantification of the 16S rRNA of the total bacteria in the gut of the larvae (mean ± SE). The soya, OBRlow, and OBRhigh treatments contained only soybean meal, a low amount of OBR, and a high amount of OBR, respectively, 1, 3, and 6 represent the first, third, and sixth day, respectively.

The abundance of gut bacterial microbiota was affected by the cultivation time only between soya groups (*P* < 0.05, soya-1 vs. soya-3 vs. soya-6) and affected by the different substrates on day 3 (*P* < 0.01, soya-3 vs. OBRlow-3 vs. OBRhigh-3) (Figure 2).

After high-throughput sequencing and bioinformatics analysis, a total of 938,102 high-quality sequences were obtained, and 1,440 OTUs were identified.

**FIGURE 3:**
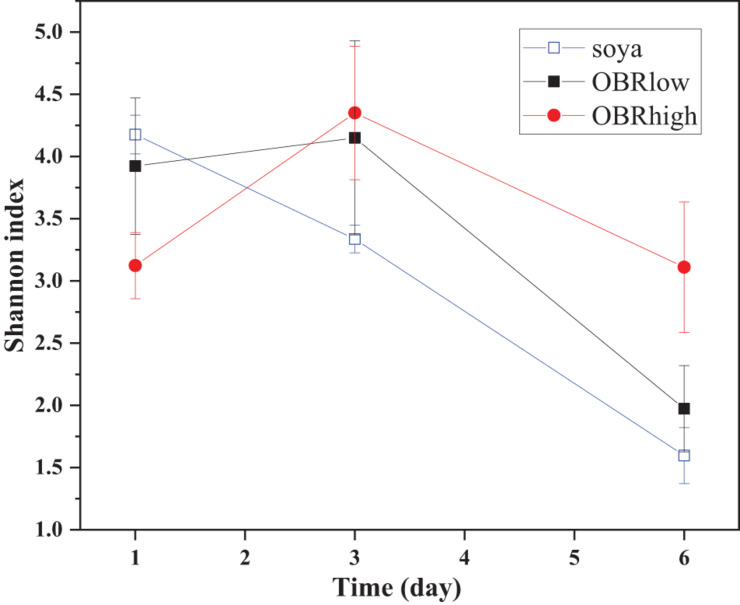
The community diversity (mean ± SE) of gut bacteria. The soya, OBRlow, and OBRhigh treatments contained only soybean meal, low amounts of OBR, and high amounts of OBR, respectively, 1, 3, and 6 represent the first, third, and sixth day, respectively.

It was obvious that the diversity of the larval gut bacterial community was affected by the different substrates and cultivation time (Figure 3 and Supplementary Figure 3). Without OBR in the substrate (soya groups), the effect of cultivation time was most significant (*P* < 0.001), while as the relative amount of OBR in the substrate increased, the differences in the effect of the cultivation time decreased (*P* < 0.05 in the OBRlow groups and no significant differences in the OBRhigh groups). Although the levels of OBR in the substrate were different, on the first and third days, there were no significant effects on diversity, while on the sixth day, the diversity was significantly affected (*P* < 0.05).

PCoA provided a visualization of the changes in the gut bacterial community structure affected by the substrates and cultivation time (Supplementary Figure 4). A clear separation appeared among the different cultivation times (PCA axis 1), which accounted for 72.03% of all variability among samples. On the first and sixth days, the various samples clustered together, and the separation among the day 1 and 6 groups was most obvious. However, for the day 1 groups, the separation was affected by the different substrates. This result indicated that the cultivation time determined the largest separation between bacterial communities (PC 1), and this phenomenon was consistent with the community diversity.

#### Composition of the Larval Bacterial Community

**FIGURE 4:**
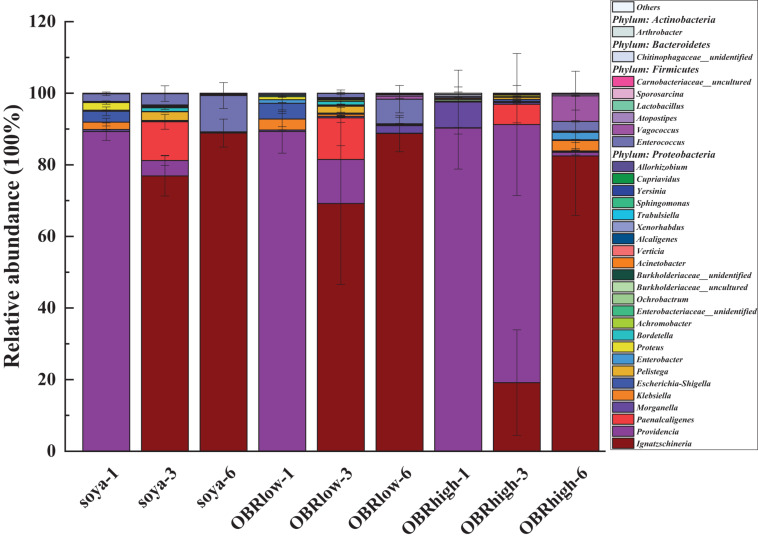
The genus-level composition (mean, *n* = 3) of the larval gut microbiota. Only the most abundant taxa (>0.1% genus) are shown (mean, *n* = 3). The soya, OBRlow, and OBRhigh represent treatments containing only soybean meal, a low amount of OBR, and a high amount of OBR, respectively, 1, 3, and 6 represent the first, third, and sixth day, respectively.

The larval gut bacterial community structure and composition were altered by the cultivation time and different substrates containing OBR (Figure 4 and Supplementary Figures 5, 6). The relative abundance of *Proteobacteria* was highest in all gut samples, and the relative abundance levels of *Firmicutes* and *Actinobacteria* were lower for substrates with OBR mixed than for those without OBR mixed (Supplementary Figure 5). *Proteobacteria* and *Firmicutes* were dominant and might represent the main bacteria involved in larval development and OBR digestion.

Among the different larval samples, *Ignatzschineria* (as high as 88.9%), *Providencia* (as high as 90.2%), and *Enterococcus* (as high as 10.1%) in the larval gut were affected significantly by the different substrates and cultivation times, with higher relative abundance levels than other genera (Figure 4 and Supplementary Figure 6). These genera varied in their relative abundance and in their response to the different substrates and time, and they might be the main representative bacteria driving the variation in gut bacterial diversity. The relative abundance levels of *Ignatzschineria* (*P* < 0.001), *Enterococcus* (of soya groups, *P* < 0.05), and *Vagococcus* increased; *Providencia* (*P* < 0.001) decreased notably over the cultivation time. *Morganella*, *Ignatzschineria* (of day three groups, *P* < 0.05), *Providencia* (of day three groups, *P* < 0.01), and *Vagococcus* varied in their relative abundance and in their responses to the different substrates.

### ARGs and MGEs in the Larval Gut

#### Diversity of ARGs and MGEs

**FIGURE 5:**
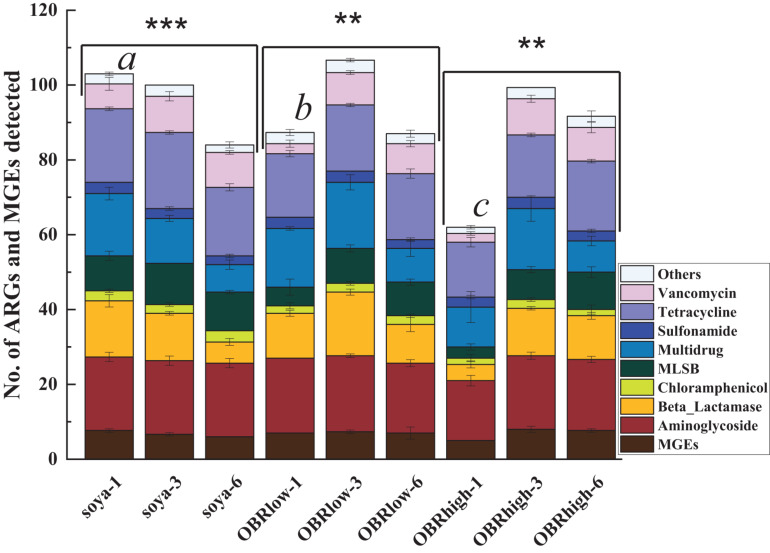
Numbers of detected ARGs and MGEs (mean ± SE, *n* = 3). An ARG was considered detected when it was amplified in one sample; because many resistance genes were targeted by multiple primers, the detection of the same gene by multiple primer sets was counted as the detection of only a single unique resistance gene. “^∗∗^” and “^∗∗∗^” indicate *P* < 0.01 (among OBRlow groups and OBRhigh groups) and *P* < 0.001 (among soya groups), respectively. Different letters indicate significant differences (*P* < 0.01, soya-1 vs. OBRlow-1 vs. OBRhigh-1). The soya, OBRlow, and OBRhigh represent treatments containing only soybean meal, a low amount of OBR, and a high amount of OBR, respectively, 1, 3, and 6 represent the first, third, and sixth day, respectively.

In the larval gut, 164 genes were detected, which included 153 abundant and diverse ARGs, 16S rRNA genes, and all MGEs (Figure 5). The numbers of detected ARGs were similar among the samples and ranged from 57 to 100, with an average of 84. On the first day, the numbers of detected ARGs and MGEs decreased as the relative amount of OBR increased compared with that without OBR mixed, and it was affected significantly by the different substrates (*P* < 0.01) (soya-1 vs. OBRlow-1 vs. OBRhigh-1). However, on the third and sixth days, compared with the treatment containing only soybean meal, when the substrates were mixed with OBR, the numbers of detected ARGs and MGEs increased slightly, and the numbers of detected ARGs and MGEs were significantly influenced by the cultivation time. In the treatment containing only soybean meal, the numbers of detected ARGs and MGEs decreased significantly with the extension of cultivation time. Conversely, in the treatment containing a low amount of OBR and the treatment containing a high amount of OBR, the numbers increased from day 1 to 3 and decreased from day 3 to 6.

All the detected ARGs represented all major resistance mechanisms, including antibiotic deactivation, efflux pumping, and cellular protection (Supplementary Figure 7A). Unique MGEs, such as *cIntI-1 (class 1), intI-1 (clinical), tnpA-01, tnpA-02, tnpA-04*, and *tnpA-05*, were detected in all 9 groups of triplicate samples, even though they varied among the different samples. In addition, based on the antibiotic to which they conferred resistance, all the detected ARGs in the larval guts could be classified into 9 types (Supplementary Figure 7B) and conferred resistance to almost all major classes of antibiotics commonly administered to humans and animals (Zhu et al., 2013, 2017). Of all the detected ARGs and MGEs in the larval gut, 29 ARGs (including 10 tetracycline resistance genes, 11 aminoglycoside resistance genes, 2 beta-lactam resistance genes, 1 MLSB resistance gene, 3 multidrug resistance genes, 1 sulfonamide resistance gene, and 2 vancomycin resistance genes) were found in all samples and represented diverse resistance mechanisms: antibiotic inactivation (14 ARGs), efflux pump (11 ARGs), and cellular protection (4 ARGs).

#### Abundance of ARGs and MGEs

**FIGURE 6:**
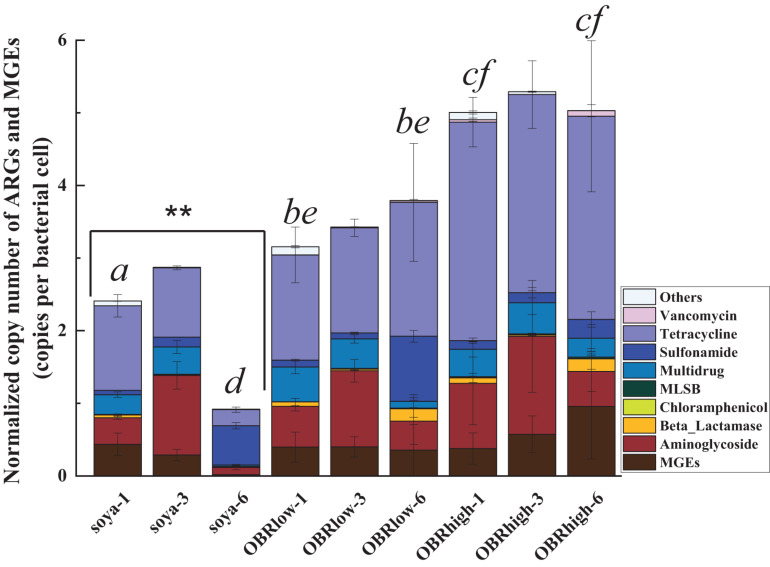
Abundance of ARGs and MGEs in the guts of larvae. Normalized copy numbers of ARGs and MGEs (mean ± SE, *n* = 3). “^∗∗^” indicates *P* < 0.01 (among soya groups). Different letters indicate significant differences (*P* < 0.05, soya-1 vs. OBRlow-1 vs. OBRhigh-1, and soya-6 vs. OBRlow-6 vs. OBRhigh-6). The soya, OBRlow and OBRhigh represent treatments containing only soybean meal, a low amount of OBR and a high amount of OBR, respectively, 1, 3, and 6 represent the first, third, and sixth day, respectively.

Of all the detected ARGs and MGEs, the absolute copy numbers of each sample ranged from 0.25 × 10^7^ to 2.87 × 10^7^ copies g^–1^ (dry weight of gut) (Supplementary Figure 8). The normalized copy numbers of ARGs and MGEs were employed to minimize potential variations in background bacterial abundances (Liu et al., 2020; Figure 6).

The normalized copy numbers of all the detected ARGs and MGEs ranged from 0.92 to 2.87 copies per bacterial cell in the treatment containing only soybean meal, from 3.16 to 3.79 copies per bacterial cell in the treatment containing a low amount of OBR, and from 5.01 to 5.29 copies per bacterial cell in the treatment containing a high amount of OBR. Notably, the abundances showed an increasing and then decreasing trend, being highest on day 3. This phenomenon was positively correlated with the consumption of substrate and the growth rate of the larvae, which also started to increase on day 3. Although only oxytetracycline antibiotics were contained in the OBR at high concentrations, the abundances of aminoglycoside resistance genes, multidrug resistance genes, sulfonamide resistance genes, and tetracycline resistance genes were also detected and enriched. When OBR was added and the percentage was increased, the absolute gene copy number decreased.

However, the normalized copy numbers of tetracycline resistance genes accounted for a fairly large proportion, from 42.56 to 63.33%, in the treatments containing low amounts of OBR and high amounts of OBR; in particular, with the increase in concentration of oxytetracycline, the proportion of tetracycline resistance genes also gradually increased (Supplementary Figure 9).

The presence and absence of gene targets of all the detected ARGs and MGEs and the degrees of the ARGs and MGEs across all samples were examined to compare the distinct patterns of resistance genes among the samples (Supplementary Figures 10, 11). As the resistance gene profiles indicated, aminoglycoside resistance genes, multidrug resistance genes, tetracycline resistance genes, and MGEs were enriched to various extents on day 3. Regarding tetracycline resistance genes, the unique *ARGs tetA-02, tetB-01, tetB-02, tetC-01, tetC-02, tetD-01, tetD-02, tetG-01, tetG-02, tetJ, tetL-02, tetM-01, tetM-02, tetQ, tetR-02*, and *tetR-03* were enriched. Moreover, the abundance of ARGs was significantly correlated with that of both integron integrase genes and transposases (Supplementary Figure 12).

### Prediction of Gut Bacterial Function

**FIGURE 7:**
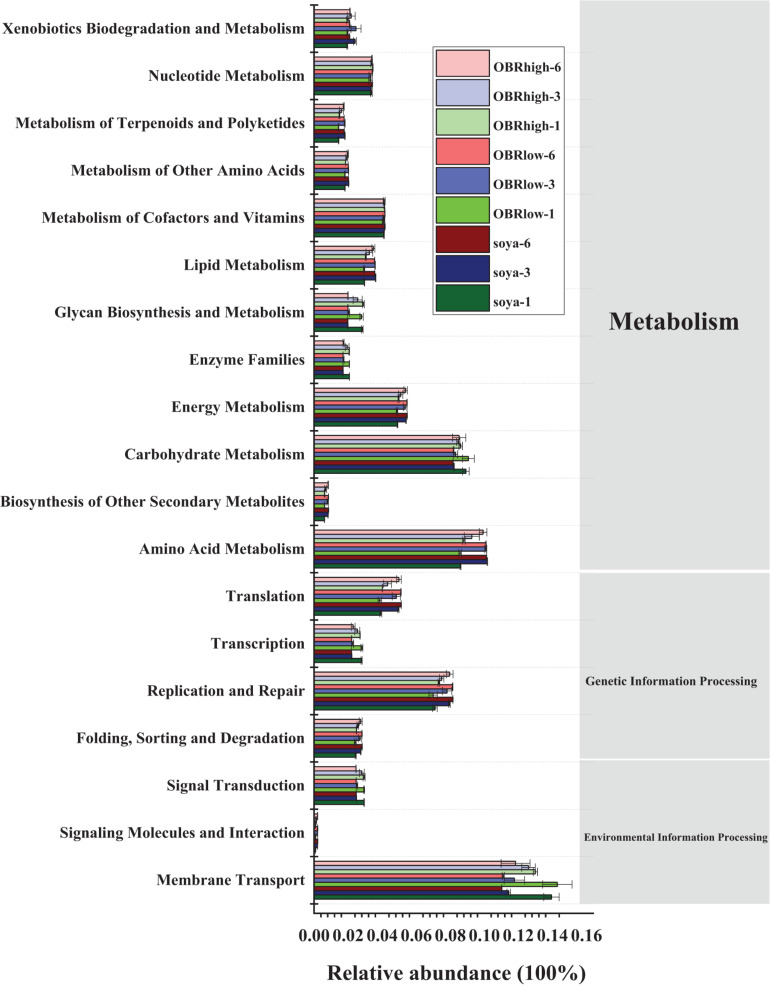
Predicted functions of the gut bacterial community in the KEGG pathway analysis performed using PICRUSt based on the KEGG database (mean *n* = 3). The soya, OBRlow, and OBRhigh represent treatments containing only soybean meal, a low amount of OBR, and a high amount of OBR, respectively, 1, 3, and 6 represent the first, third, and sixth day, respectively.

The potential functional profiles of the bacterial community in the gut were analyzed based on KEGG pathways. In all samples (27 samples) (Figure 7), metabolism (43.50–45.63%) was the major predicted function, followed by genetic information processing (16.08–18.28%) and environmental information processing (13.71–17.31%). Within the metabolism cluster, the main pathways were attributed to amino acid metabolism, carbohydrate metabolism, energy metabolism, metabolism of cofactors and vitamins, nucleotide metabolism, lipid metabolism, xenobiotic biodegradation, and metabolism. Within the genetic information processing cluster, the main pathways were attributed to replication and repair, translation, folding, sorting, and degradation. Within the environmental information processing cluster, the main pathways were attributed to membrane transport and signal transduction (Supplementary Table 2). Although the relative abundance of these main metabolism types varied slightly depending on the substrate and cultivation time, these types retained a high relative abundance in all samples. In particular, the relative abundance of ABC transporters, a bacterial secretion system, the phosphotransferase system (PTS) and transporters within the membrane transport cluster remained relatively high in all samples. Moreover, within the xenobiotic biodegradation and metabolism cluster, the functions included the degradation of toxic and harmful organic pollutants, such as aminobenzoate, benzoate, bisphenol, caprolactam, chloroalkane, dioxin, nitrotoluene, polycyclic aromatic hydrocarbons, styrene, toluene, and xylene.

## Discussion

Most of the larvae escaped from the substrates when the OBR content was high, and toxic components, such as byproducts, metabolic products, and especially oxytetracycline residues (Guo et al., 2012), which remained in the OBR may exceed the selection pressure on larvae to digest and convert substates. The larvae escaped when the content of OBR was 100 and 50% in the substrates uniformly mixed with soya meal, whereas the larvae could digest the mixed substrates when the OBR:soya bean ratio was 1:2. Therefore, the highest larval selection pressure to oxytetracycline may range from 3,042 to 4,653 mg kg^–1^ dry weight, which is well above the concentration of tetracycline remaining in almost all organic wastes or the environment, except for OBR (Xie et al., 2016). When the substrates were mixed with OBR and soybean meal, the larvae gained weight and consumed 70.8, 59.3, and 54.5% of the substrate in the treatments containing only soybean meal, a low amount of OBR, and a high amount of OBR, respectively. With OBR addition to the substrate, the larvae lost weight, but larval mortality was not examined. Therefore, larval reproductive and other fitness trait impacts should be measured to better determine larval stress tolerance and ability. After the larvae migrated and were harvested from the substrate, 65.9 to 63.3% of the oxytetracycline was effectively degraded in the treatments containing low amounts of OBR and high amounts of OBR, respectively, and no oxytetracycline was detected in the larvae. However, whether oxytetracycline accumulates in larval tissue needs to be further determined and verified (Lalander et al., 2016). This phenomenon may represent a content of oxytetracycline that was not within the detectable range; some oxytetracycline remained in the lipid fraction and was difficult to extract and determine, or it did not accumulate in the larval body, similar to other organic compounds (Purschke et al., 2017). It is effective to mix OBR with other organic matter or organic waste as substrates for degradation by black soldier flies because of the large numbers of oxytetracycline residues, heavy metals, fermentation byproducts, and metabolic products remaining in the bacterial residues (Guo et al., 2012). Therefore, the nutrients in substrates and natural ecological factors, such as parasites, microorganisms, organic and inorganic compounds, and salt, may affect the growth and development of these larvae (Camenzuli et al., 2018; Meneguz et al., 2018; Liu et al., 2019). Therefore, in this treatment, the residuum remaining in the OBR may affect the ability of larvae to degrade oxytetracycline. Although the degradation efficiency was slightly lower than in previous results of 82.7% (Liu et al., 2020), this disposal by larvae may be an economical, secure, and effective method compared with composting and anaerobic digestion, among others (Kakimoto and Funamizu, 2007; Kakimoto et al., 2007; Zhou et al., 2011; Li et al., 2012; Zhou et al., 2012). If the larvae were domesticated, they might be better able to adapt to complex environments, degrade the oxytetracycline remaining in OBR, and convert the OBR.

The gut microbiome plays an important role in the conversion of organic waste (Nguyen et al., 2015; De Smet et al., 2018; Liu et al., 2019) and varies with substrates (Jeon et al., 2011). In particular, the growth of a variety of sensitive microbes might be inhibited by antibiotics, while tolerant microbes adapt quickly and survive as the environment changes (Selvam et al., 2012). The oxytetracycline remaining in the OBR is a wide-spectrum bactericide and is expected to disturb multiple members of the gut microbiota. Due to consumption by the larvae and gut microbiota, the available nutrient contents in the substrates decrease, and the combination of different nutrients and oxytetracycline in the substrate may lead to differences in abundance of the gut microbiota. However, to maintain gastrointestinal health, the intestinal ecosystem must have sufficient functional redundancy or strong resilience via the formation of resistance to oxytetracycline or self-protection. Therefore, larvae may adapt to their environment by adjusting their own adaptability when the environmental systems are complex. Notably, the abundance, structure, and composition of the gut microbiota differed on the basis of the substrate composition and over time. More interestingly, the abundance of the gut microbiota indicated that bacterial biomass was decreasing in the larval guts on day 6 and was most striking with the highest OBR treatment. However, the diversity of the gut microbiota in the high OBR treatment was higher than other treatments on day 6 which is displayed in Figure 3, and further displayed in Figure 4 with the relative abundance of the taxa. Therefore, while the total bacterial abundance decreased with increasing OBR, the highest content of OBR was enriched in targeted and most likely resistant microbes, and the observed shifts in bacterial composition might contribute to the adaptation and degradation of OBR. Based on the bioinformatics analysis of high-throughput sequencing data, *Enterococcus, Ignatzschineria, Providencia, Morganella, Paenalcaligenes*, and *Proteus* in the larval gut might be the dominant genera contributing to degradation and consumption of the OBR by larvae. Although the structure and composition were slightly different from those of other organic wastes, the species of intestinal microbiota from the phylum through the genus level were similar to those reported previously (Bruno et al., 2019; Liu et al., 2020). This difference may be due to the significant variation of the larval gut bacterial communities from the substrates (Bruno et al., 2019). Additionally, variation in the composition of the gut bacterial communities further affected the differences in biological characteristics of black soldier fly larvae (Bonelli et al., 2019; Wynants et al., 2019). Compared with the communities in the guts of other insects, the identified communities in this larval gut are unique (De Smet et al., 2018). These identified and unique communities in the larval gut might be the key for larvae to consume and degrade complex organic waste based on specific metabolic properties (Warnecke et al., 2007), ensuring larval survival and OBR degradation (Liu et al., 2019).

As reported, HGT (horizontal gene transfer) mechanisms are a major cause of resistance gene transfer when ARGs associated with MGEs move from one species to another with easy dissemination and strong persistence in various environments. The abundance of ARGs was significantly correlated with that of both integron-integrase genes and transposases. This result suggested that gene transfer occurred during transport, and different types of ARGs remained in larval guts. Therefore, abundant and varied ARGs have been discovered in the gut. Although the presence of antibiotics and the microbial community were the major determinants shaping the gut antibiotic resistome by phylogeny (Forsberg et al., 2012) and only oxytetracycline antibiotics were contained in the OBR at high concentrations, many other types of ARGs may derive from environmental non-pathogenic sources (Stokes and Gillings, 2011; Wang et al., 2015) or the HGT (Gorokhova et al., 2015) of MGEs (plasmids, integrons, transposons, etc.) (D’costa et al., 2006; Gaze et al., 2011; Babakhani and Oloomi, 2018). Alternatively, other types of ARGs may be affected by the structure of the microbial community (Forsberg et al., 2014). As a result, when the relative content of OBR increased, the abundance of ARGs increased, which suggests that gut microbes are potentially activating these genes to increase tolerance and resistance in the substrates with increasing OBR. This inadaptability was clearly demonstrated with the substrates of 1:1 OBR:soya meal, from which most of the larvae escaped. As previously shown, *Enterococcus* (Lauderdale et al., 2007), and *Bacillus* (D’costa et al., 2006) are generally recognized as related to antibiotic resistance. In particular, concerning the predicted source of antibiotic resistance, *Proteobacteria* are prominent (Forsberg et al., 2014) and have been demonstrated to be potential hosts of tetracycline resistance genes (Jang et al., 2018). *Firmicutes, Proteobacteria, Actinobacteria*, and *Bacteroidetes* in the guts of larvae clearly were all potential hosts of ARGs, as *Enterococcus, Ignatzschineria, Bordetella, Providencia*, and *Proteus* were significantly correlated with tetracycline resistance genes and considered to be potential hosts of tetracycline resistance genes (Liu et al., 2020). In this analysis, the proportions of tetracycline resistance genes were higher with OBR than without OBR, which indirectly indicated self-protection and the formation of resistance to oxytetracycline by the gut microbiota. Although the types of ARGs and their abundances varied among the samples due to the use of different substrates, all the major resistance mechanisms can be represented by these detected and abundant ARGs. This finding indicated that the larvae could adapt to environmental conditions with OBR as a substrate because of the diverse and abundant ARGs in their gut. Therefore, OBR could be degraded and utilized, and the residual oxytetracycline could be degraded by the larvae.

Microorganisms can degrade contaminants for ecosystem self-purification processes based on metabolic and/or cometabolic pathways (Grenni et al., 2018), and the metabolic capacity and level of the microbiotas determine the efficiency of degradation, recycling, and utilization of organic matter. Importantly, evidence shows that in the gut of insects, the metabolic reactions of organic matter are not encoded by the larval genome but by the metagenome of the larval gut microbiota (De Smet et al., 2018). Furthermore, biodegradation of microorganisms is considered the most critical process in eliminating the majority of xenobiotics (Kelsic et al., 2015). Biodegradation based on antibiotic-resistant bacteria and ARGs is the key to the effective degradation of antibiotics in the environment (Dantas et al., 2008). The bacterial community was identified as the major driver for the distribution and abundance of ARGs (Forsberg et al., 2014). In particular, because the intestinal bacteria of animals promote the widespread dissemination of ARGs, the guts of animals have been recognized as a particularly important reservoir in the environment (He et al., 2016). In addition to abundant and varied ARGs, abundant and highly active digestive enzymes were also observed in the larval gut. Thus, the larval gut microbiome played an important role in the degradation of oxytetracycline and the consumption of OBR. The analysis of the potential gut bacterial function showed that metabolism was the major predicted function, followed by genetic information processing and environmental information processing. Amino acid metabolism, carbohydrate metabolism, energy metabolism, metabolism of cofactors and vitamins, nucleotide metabolism, lipid metabolism, and xenobiotic biodegradation and metabolism were the main pathways for metabolism, which ensured the metabolism and utilization of organic matter in the substrate. Folding, sorting, and degradation; replication and repair; transcription; and translation within genetic information processing were recovered functions, which ensured the survival of larvae in harsh environmental conditions (Lukhele et al., 2019). In particular, the high relative abundance of ABC transporters, bacterial secretion system, phosphotransferase system (PTS), secretion system, and transporters within the membrane transport cluster ensured the adaptation and degradation ability of larvae. These results indicated that the gut bacterial community could respond to the changing environment, implying a relatively stable survival and adaptation strategy during degradation, and appearing to promote the production of energy and degradation of organics in substrates by the larvae. More importantly, within the xenobiotic biodegradation and metabolism cluster, the functions included the degradation of toxic and harmful organic pollutants, such as aminobenzoate, benzoate, bisphenol, caprolactam, chloroalkane, dioxin, nitrotoluene, polycyclic aromatic hydrocarbons, styrene, toluene, and xylene. This result further indicated that the larvae could degrade hazardous organic pollutants with phenyl rings, which might be indirect evidence that the larvae can degrade oxytetracycline and utilize OBR for energy. Therefore, based on the predicted functions of the gut bacterium, the oxytetracycline remaining in the OBR and the OBR itself could be degraded and utilized by the larvae.

The larvae clearly could convert OBR into organic matter for the insect body, and the oxytetracycline could be degraded effectively by the gut bacterium. Generally, within the tolerance range of the larvae or their intestinal bacterium, the larvae could utilize the OBR and degrade residual oxytetracycline effectively based on the resistance of the intestinal bacterium to oxytetracycline and the ability of the larvae to degrade organic waste. In particular, the cultivation time was the main factor affecting the conversion and degradation of the substrate by the larvae. Therefore, black soldier fly larvae are an effective bioremediation tool for the treatment and utilization of antibiotic bacterial residues. Regrettably, in this research, the degradation behavior of the parent compounds was the focus. However, the degradation products or metabolites of oxytetracycline depend on different environmental and non-environmental factors because the molecular structure possesses multiple ionizable functional groups, so the toxicity and trend of antibiotic degradation products or metabolites degraded by black soldier fly larvae are unknown. Thus, although the OBR was rich in high-quality protein, fat, cellulose, enzymes, and other components, it was regarded as a unique hazardous waste due to its complex composition, which consisted of large numbers of oxytetracycline residues, heavy metals, fermentation byproducts, metabolic products and byproducts. Therefore, whether larvae could be used as a value-added resource is unknown; how to eliminate toxicity and potential harm completely and effectively has not been confirmed.

This is the first report to examine the digestive physiology of black soldier fly larvae to dispose of and degrade oxytetracycline remaining in the OBR. In addition, the oxytetracycline remaining in the OBR could be degraded efficiently. However, the natural degradation of OBR within the substrate without the addition of larvae was not examined, which could be a limitation and make it difficult to compare the background degradation levels to the efficiency of larval microbiome degradation. In addition, after utilization of the OBR, almost all ARGs were detected in high abundance, even though only tetracycline antibiotics remained in the OBR. The gut is an important site for the accumulation of ARGs (Hu et al., 2013), which are regarded as emerging contaminants posing a potential worldwide human health risk (Zhu et al., 2013, 2017). The enrichment of ARGs and MGEs in the guts of larvae must be considered, and the degradation and removal of accumulated ARGs in the larval gut may be a challenge.

## Conclusion

The hazardous waste OBR can be digested by black soldier fly larvae. Incidentally, the oxytetracycline remaining in the OBR can also be effectively degraded, potentially mainly due to the metabolic capacity and characteristics of larval intestinal bacteria. Hence, larval degradation could be one part of a bioremediation strategy, and further research on the process and underlying mechanism is needed to achieve the safe and efficient bioremediation of antibiotic bacterial residue by black soldier fly larvae.

## Data Availability Statement

This sequencing data can be found at: https://www.ncbi.nlm.nih.gov/bioproject/PRJNA640797.

## Author Contributions

CL: investigation, visualization, data curation, and writing–original draft. HY and CW: conceptualization, methodology, supervision, writing–review, editing, project administration, and validation. All authors contributed to the article and approved the submitted version.

## Conflict of Interest

The authors declare that the research was conducted in the absence of any commercial or financial relationships that could be construed as a potential conflict of interest.
