# Supplementary material for: Black Soldier Fly Larvae Can Effectively Degrade Oxytetracycline Bacterial Residue by Means of the Gut Bacterial Community
Source: Front Microbiol. 2021 Jun 15;12:663972. doi: 10.3389/fmicb.2021.663972 (PMC8239407; doi:10.3389/fmicb.2021.663972)
Supplement: Supplementary file 1 [file Data_Sheet_1.docx]

# Supporting Material for

**Black soldier fly larvae can effectively degrade oxytetracycline bacterial residue by means of the gut bacterial community**

Cuncheng Liu,^1,2^ Huaiying Yao, ^1,3,4,*^ Cunwen Wang, ^2,*^

^1^ Research Center for Environmental Ecology and Engineering, School of Environmental Ecology and Biological Engineering, Wuhan Institute of Technology, Wuhan 430073, People's Republic of China

^2^ Key Laboratory of Green Chemical Process of Ministry of Education, Key Laboratory of Novel Reactor and Green Chemical Technology of Hubei Province, School of Chemical Engineering and Pharmacy, Wuhan Institute of Technology, Wuhan 430205, People's Republic of China

^3^ Zhejiang Key Laboratory of Urban Environmental Processes and Pollution Control, Ningbo Urban Environment Observation and Research Station, Chinese Academy of Sciences, Ningbo 315800, People's Republic of China

^4^ Key Laboratory of Urban Environment and Health, Institute of Urban Environment, Chinese Academy of Sciences, Xiamen, 361021, People's Republic of China

* Corresponding authors:

E-mail addresses: [hyyao@iue.ac.cn](mailto:hyyao@iue.ac.cn) (H.Yao), [wangcw0120@163.com](mailto:wangcw0120@163.com) (C. Wang).

# Contents

## Tables

Table 1: *P*-values of repeated measures ANOVA on the effects of different pretreatment.

Table 2: Prediction of gut microbial function by Phylogenetic Investigation of Communities by Reconstruction of Unobserved States (PICRUSt). Data are relative abundances (100%).

## Equations

Equation 1: Residual solids digested by larvae.

Equation 2: Consumption of diet.

Equation 3: Expected concentration of oxytetracycline in the diet at any time.

Equation 4: Degradation efficiency and residual oxytetracycline.

## Figures

Figure 1. The diagram of dried oxytetracycline bacterial residue.

Figure 2: Changes in (a) the consumption of substrate and (b) the fresh weight of larvae.

Figure 3: Rarefaction curves of all the samples (label: 0.03).

Figure 4: Principal coordinate analysis (PCoA) of larval gut samples.

Figure 5: The gut microbiota at the phylum level.

Figure 6: Heat maps showing the enrichment of taxa.

Figure 7: (a) Proportions of total antibiotic resistance genes (ARGs) detected in the gut classified by the resistance mechanism. (b) Proportions of total ARGs detected in the gut classified by the resistance to antibiotics.

Figure 8: Abundance of ARGs and MGEs in the guts of larvae.

Figure 9: Normalized copy numbers of tetracycline resistance gene occupancy.

Figure 10: The profile of MGEs and ARGs that confer resistance to each class of antibiotics.

Figure 11: Gene profiles of ARGs and MGEs detected in the guts of larvae.

Figure 12: Correlation of the normalized gene copy numbers of ARGs with the normalized copy number of transposases or of the class 1 integron-integrase gene.

## Total:

Number of tables: 2

Number of equations: 4

Number of figures: 12

Number of pages: 24

# Tables

Table 1. *P*-values of repeated measures ANOVA on the effects of different pretreatment. The soya, OBRlow and OBRhigh represented treatments containing only soybean meal, low amount of OBR and high amount of OBR, respectively.

|  |  | Time (day) | | | | | | |
| --- | --- | --- | --- | --- | --- | --- | --- | --- |
|  |  | 1 | 2 | 3 | 4 | 5 | 6 | 7 |
| soya vs | OBRlow | 0.0596 | 0.0161 | 0.0888 | 0.1001 | 0.0022 | 0.0167 | 0.0087 |
|  | OBRhigh | 0.0055 | 0.0020 | 0.0067 | 0.1168 | 0.0001 | 0.0003 | 0.0004 |
| OBRlow vs | OBRhigh | 0.1560 | 0.0052 | 0.8977 | 0.8905 | 0.0002 | 0.0010 | 0.0005 |

## Table 2. Prediction of gut microbial function by Phylogenetic Investigation of Communities by Reconstruction of Unobserved States (PICRUSt). Data are relative abundances (100%).The soya, OBRlow and OBRhigh represented treatments containing only soybean meal, low amount of OBR and high amount of OBR, respectively; 1, 3, 6 represented the first day, third day and sixth day, respectively.

| level 1 | level 2 | level 3 | soya-1 | soya-3 | soya-6 | OBRlow-1 | OBRlow-3 | OBRlow-6 | OBRhigh-1 | OBRhigh-3 | OBRhigh-6 |
| --- | --- | --- | --- | --- | --- | --- | --- | --- | --- | --- | --- |
| Environmental Information Processing | Membrane Transport | ABC transporters | 0.0372 | 0.0278 | 0.0255 | 0.0383 | 0.0292 | 0.0257 | 0.0348 | 0.0328 | 0.0279 |
|  |  | Bacterial secretion system | 0.0096 | 0.0127 | 0.0130 | 0.0096 | 0.0124 | 0.0130 | 0.0093 | 0.0106 | 0.0126 |
|  |  | Phosphotransferase system (PTS) | 0.0066 | 0.0012 | 0.0011 | 0.0071 | 0.0016 | 0.0013 | 0.0050 | 0.0038 | 0.0028 |
|  |  | Secretion system | 0.0263 | 0.0299 | 0.0307 | 0.0261 | 0.0294 | 0.0305 | 0.0263 | 0.0278 | 0.0295 |
|  |  | Transporters | 0.0596 | 0.0428 | 0.0400 | 0.0617 | 0.0450 | 0.0406 | 0.0546 | 0.0511 | 0.0455 |
|  | Signal Transduction | Calcium signaling pathway | 0.0000 | 0.0000 | 0.0000 | 0.0000 | 0.0000 | 0.0000 | 0.0000 | 0.0000 | 0.0000 |
|  |  | MAPK signaling pathway - yeast | 0.0003 | 0.0004 | 0.0004 | 0.0003 | 0.0004 | 0.0004 | 0.0003 | 0.0003 | 0.0004 |
|  |  | Phosphatidylinositol signaling system | 0.0012 | 0.0013 | 0.0014 | 0.0012 | 0.0013 | 0.0014 | 0.0013 | 0.0013 | 0.0014 |
|  |  | Two-component system | 0.0279 | 0.0232 | 0.0229 | 0.0280 | 0.0235 | 0.0229 | 0.0278 | 0.0264 | 0.0229 |
|  | Signaling Molecules and Interaction | Bacterial toxins | 0.0001 | 0.0010 | 0.0011 | 0.0001 | 0.0009 | 0.0010 | 0.0000 | 0.0003 | 0.0010 |
|  |  | Cellular antigens | 0.0005 | 0.0011 | 0.0011 | 0.0005 | 0.0010 | 0.0010 | 0.0005 | 0.0007 | 0.0010 |
|  |  | Ion channels | 0.0003 | 0.0000 | 0.0000 | 0.0003 | 0.0000 | 0.0000 | 0.0003 | 0.0002 | 0.0000 |
| Genetic Information Processing | Folding, Sorting and Degradation | Chaperones and folding catalysts | 0.0109 | 0.0123 | 0.0127 | 0.0109 | 0.0121 | 0.0126 | 0.0111 | 0.0115 | 0.0123 |
|  |  | Proteasome | 0.0003 | 0.0003 | 0.0004 | 0.0002 | 0.0003 | 0.0004 | 0.0003 | 0.0003 | 0.0003 |
|  |  | Protein export | 0.0043 | 0.0055 | 0.0056 | 0.0043 | 0.0053 | 0.0056 | 0.0044 | 0.0048 | 0.0055 |
|  |  | Protein processing in endoplasmic reticulum | 0.0003 | 0.0007 | 0.0007 | 0.0002 | 0.0006 | 0.0007 | 0.0003 | 0.0004 | 0.0006 |
|  |  | RNA degradation | 0.0038 | 0.0048 | 0.0049 | 0.0037 | 0.0046 | 0.0049 | 0.0039 | 0.0042 | 0.0048 |
|  |  | Sulfur relay system | 0.0046 | 0.0038 | 0.0039 | 0.0046 | 0.0039 | 0.0039 | 0.0046 | 0.0043 | 0.0039 |
|  |  | Ubiquitin system | 0.0003 | 0.0000 | 0.0000 | 0.0003 | 0.0000 | 0.0000 | 0.0003 | 0.0002 | 0.0000 |
|  | Replication and Repair | Base excision repair | 0.0034 | 0.0041 | 0.0042 | 0.0034 | 0.0041 | 0.0042 | 0.0034 | 0.0037 | 0.0042 |
|  |  | Chromosome | 0.0140 | 0.0159 | 0.0162 | 0.0138 | 0.0157 | 0.0162 | 0.0142 | 0.0148 | 0.0159 |
|  |  | DNA repair and recombination proteins | 0.0238 | 0.0261 | 0.0268 | 0.0235 | 0.0257 | 0.0268 | 0.0246 | 0.0249 | 0.0262 |
|  |  | DNA replication | 0.0052 | 0.0055 | 0.0056 | 0.0050 | 0.0054 | 0.0056 | 0.0054 | 0.0054 | 0.0055 |
|  |  | DNA replication proteins | 0.0093 | 0.0096 | 0.0099 | 0.0091 | 0.0095 | 0.0099 | 0.0096 | 0.0095 | 0.0097 |
|  |  | Homologous recombination | 0.0072 | 0.0081 | 0.0085 | 0.0071 | 0.0080 | 0.0084 | 0.0075 | 0.0076 | 0.0082 |
|  |  | Mismatch repair | 0.0062 | 0.0068 | 0.0071 | 0.0060 | 0.0067 | 0.0070 | 0.0064 | 0.0065 | 0.0068 |
|  |  | Non-homologous end-joining | 0.0000 | 0.0004 | 0.0004 | 0.0000 | 0.0003 | 0.0003 | 0.0000 | 0.0001 | 0.0003 |
|  |  | Nucleotide excision repair | 0.0020 | 0.0028 | 0.0028 | 0.0020 | 0.0027 | 0.0028 | 0.0021 | 0.0023 | 0.0027 |
|  | Transcription | Basal transcription factors | 0.0000 | 0.0000 | 0.0000 | 0.0000 | 0.0000 | 0.0000 | 0.0000 | 0.0000 | 0.0000 |
|  |  | RNA polymerase | 0.0010 | 0.0014 | 0.0014 | 0.0010 | 0.0013 | 0.0014 | 0.0010 | 0.0012 | 0.0014 |
|  |  | Transcription factors | 0.0218 | 0.0126 | 0.0120 | 0.0221 | 0.0134 | 0.0122 | 0.0206 | 0.0180 | 0.0136 |
|  |  | Transcription machinery | 0.0051 | 0.0082 | 0.0085 | 0.0050 | 0.0079 | 0.0084 | 0.0053 | 0.0063 | 0.0080 |
|  | Translation | Aminoacyl-tRNA biosynthesis | 0.0066 | 0.0097 | 0.0099 | 0.0065 | 0.0094 | 0.0098 | 0.0069 | 0.0078 | 0.0096 |
|  |  | mRNA surveillance pathway | 0.0000 | 0.0000 | 0.0000 | 0.0000 | 0.0000 | 0.0000 | 0.0000 | 0.0000 | 0.0000 |
|  |  | Ribosome | 0.0135 | 0.0184 | 0.0191 | 0.0133 | 0.0179 | 0.0190 | 0.0139 | 0.0154 | 0.0186 |
|  |  | Ribosome Biogenesis | 0.0135 | 0.0152 | 0.0159 | 0.0133 | 0.0148 | 0.0158 | 0.0136 | 0.0141 | 0.0155 |
|  |  | Ribosome biogenesis in eukaryotes | 0.0005 | 0.0010 | 0.0011 | 0.0005 | 0.0009 | 0.0010 | 0.0005 | 0.0007 | 0.0010 |
|  |  | RNA transport | 0.0012 | 0.0010 | 0.0011 | 0.0012 | 0.0010 | 0.0011 | 0.0013 | 0.0012 | 0.0011 |
|  |  | Translation factors | 0.0038 | 0.0042 | 0.0042 | 0.0037 | 0.0041 | 0.0042 | 0.0039 | 0.0040 | 0.0042 |
| Metabolism | Amino Acid Metabolism | Alanine, aspartate and glutamate metabolism | 0.0077 | 0.0081 | 0.0081 | 0.0076 | 0.0080 | 0.0081 | 0.0079 | 0.0079 | 0.0080 |
|  |  | Amino acid related enzymes | 0.0106 | 0.0141 | 0.0145 | 0.0106 | 0.0137 | 0.0144 | 0.0107 | 0.0119 | 0.0140 |
|  |  | Arginine and proline metabolism | 0.0093 | 0.0121 | 0.0123 | 0.0093 | 0.0119 | 0.0123 | 0.0093 | 0.0102 | 0.0121 |
|  |  | Cysteine and methionine metabolism | 0.0081 | 0.0088 | 0.0092 | 0.0081 | 0.0087 | 0.0092 | 0.0078 | 0.0082 | 0.0091 |
|  |  | Glycine, serine and threonine metabolism | 0.0085 | 0.0070 | 0.0067 | 0.0085 | 0.0071 | 0.0067 | 0.0086 | 0.0080 | 0.0069 |
|  |  | Histidine metabolism | 0.0046 | 0.0056 | 0.0056 | 0.0046 | 0.0056 | 0.0056 | 0.0050 | 0.0052 | 0.0054 |
|  |  | Lysine biosynthesis | 0.0060 | 0.0066 | 0.0067 | 0.0059 | 0.0065 | 0.0067 | 0.0062 | 0.0063 | 0.0066 |
|  |  | Lysine degradation | 0.0031 | 0.0042 | 0.0039 | 0.0032 | 0.0042 | 0.0039 | 0.0033 | 0.0036 | 0.0037 |
|  |  | Phenylalanine metabolism | 0.0026 | 0.0040 | 0.0039 | 0.0027 | 0.0039 | 0.0039 | 0.0027 | 0.0031 | 0.0038 |
|  |  | Phenylalanine, tyrosine and tryptophan biosynthesis | 0.0060 | 0.0075 | 0.0078 | 0.0059 | 0.0073 | 0.0077 | 0.0061 | 0.0065 | 0.0075 |
|  |  | Tryptophan metabolism | 0.0041 | 0.0053 | 0.0049 | 0.0041 | 0.0053 | 0.0049 | 0.0043 | 0.0047 | 0.0048 |
|  |  | Tyrosine metabolism | 0.0051 | 0.0035 | 0.0032 | 0.0051 | 0.0037 | 0.0032 | 0.0054 | 0.0047 | 0.0033 |
|  |  | Valine, leucine and isoleucine biosynthesis | 0.0061 | 0.0077 | 0.0078 | 0.0061 | 0.0076 | 0.0077 | 0.0063 | 0.0068 | 0.0075 |
|  |  | Valine, leucine and isoleucine degradation | 0.0041 | 0.0073 | 0.0067 | 0.0040 | 0.0073 | 0.0067 | 0.0044 | 0.0055 | 0.0064 |
|  | Biosynthesis of Other Secondary Metabolites | beta-Lactam resistance | 0.0003 | 0.0004 | 0.0004 | 0.0003 | 0.0004 | 0.0004 | 0.0003 | 0.0003 | 0.0004 |
|  |  | Betalain biosynthesis | 0.0000 | 0.0000 | 0.0000 | 0.0000 | 0.0000 | 0.0000 | 0.0000 | 0.0000 | 0.0000 |
|  |  | Butirosin and neomycin biosynthesis | 0.0000 | 0.0003 | 0.0004 | 0.0000 | 0.0003 | 0.0003 | 0.0000 | 0.0001 | 0.0003 |
|  |  | Caffeine metabolism | 0.0000 | 0.0000 | 0.0000 | 0.0000 | 0.0000 | 0.0000 | 0.0000 | 0.0000 | 0.0000 |
|  |  | Flavone and flavonol biosynthesis | 0.0000 | 0.0000 | 0.0000 | 0.0000 | 0.0000 | 0.0000 | 0.0000 | 0.0000 | 0.0000 |
|  |  | Flavonoid biosynthesis | 0.0000 | 0.0000 | 0.0000 | 0.0000 | 0.0000 | 0.0000 | 0.0000 | 0.0000 | 0.0000 |
|  |  | Indole alkaloid biosynthesis | 0.0000 | 0.0000 | 0.0000 | 0.0000 | 0.0000 | 0.0000 | 0.0000 | 0.0000 | 0.0000 |
|  |  | Isoflavonoid biosynthesis | 0.0000 | 0.0000 | 0.0000 | 0.0000 | 0.0000 | 0.0000 | 0.0000 | 0.0000 | 0.0000 |
|  |  | Isoquinoline alkaloid biosynthesis | 0.0005 | 0.0007 | 0.0007 | 0.0005 | 0.0006 | 0.0007 | 0.0005 | 0.0006 | 0.0007 |
|  |  | Novobiocin biosynthesis | 0.0015 | 0.0017 | 0.0018 | 0.0015 | 0.0017 | 0.0018 | 0.0016 | 0.0016 | 0.0017 |
|  |  | Penicillin and cephalosporin biosynthesis | 0.0005 | 0.0004 | 0.0004 | 0.0005 | 0.0004 | 0.0004 | 0.0005 | 0.0005 | 0.0004 |
|  |  | Phenylpropanoid biosynthesis | 0.0002 | 0.0006 | 0.0007 | 0.0003 | 0.0006 | 0.0007 | 0.0001 | 0.0003 | 0.0008 |
|  |  | Stilbenoid, diarylheptanoid and gingerol biosynthesis | 0.0000 | 0.0000 | 0.0000 | 0.0000 | 0.0000 | 0.0000 | 0.0000 | 0.0000 | 0.0000 |
|  |  | Streptomycin biosynthesis | 0.0016 | 0.0027 | 0.0028 | 0.0016 | 0.0026 | 0.0028 | 0.0016 | 0.0020 | 0.0027 |
|  |  | Tropane, piperidine and pyridine alkaloid biosynthesis | 0.0015 | 0.0014 | 0.0014 | 0.0015 | 0.0014 | 0.0014 | 0.0015 | 0.0015 | 0.0014 |
|  | Carbohydrate Metabolism | Amino sugar and nucleotide sugar metabolism | 0.0100 | 0.0072 | 0.0074 | 0.0101 | 0.0073 | 0.0075 | 0.0097 | 0.0087 | 0.0080 |
|  |  | Ascorbate and aldarate metabolism | 0.0030 | 0.0015 | 0.0014 | 0.0031 | 0.0016 | 0.0014 | 0.0028 | 0.0024 | 0.0017 |
|  |  | Butanoate metabolism | 0.0096 | 0.0091 | 0.0085 | 0.0096 | 0.0093 | 0.0085 | 0.0099 | 0.0097 | 0.0085 |
|  |  | C5-Branched dibasic acid metabolism | 0.0031 | 0.0033 | 0.0032 | 0.0031 | 0.0033 | 0.0032 | 0.0031 | 0.0032 | 0.0031 |
|  |  | Citrate cycle (TCA cycle) | 0.0074 | 0.0078 | 0.0078 | 0.0074 | 0.0077 | 0.0077 | 0.0075 | 0.0076 | 0.0076 |
|  |  | Fructose and mannose metabolism | 0.0049 | 0.0037 | 0.0039 | 0.0053 | 0.0038 | 0.0040 | 0.0040 | 0.0039 | 0.0047 |
|  |  | Galactose metabolism | 0.0023 | 0.0023 | 0.0025 | 0.0025 | 0.0023 | 0.0025 | 0.0019 | 0.0019 | 0.0029 |
|  |  | Glycolysis / Gluconeogenesis | 0.0084 | 0.0087 | 0.0088 | 0.0086 | 0.0087 | 0.0089 | 0.0080 | 0.0082 | 0.0092 |
|  |  | Glyoxylate and dicarboxylate metabolism | 0.0069 | 0.0070 | 0.0067 | 0.0069 | 0.0071 | 0.0067 | 0.0071 | 0.0071 | 0.0066 |
|  |  | Inositol phosphate metabolism | 0.0012 | 0.0018 | 0.0018 | 0.0013 | 0.0017 | 0.0018 | 0.0011 | 0.0013 | 0.0018 |
|  |  | Pentose and glucuronate interconversions | 0.0036 | 0.0022 | 0.0021 | 0.0039 | 0.0024 | 0.0022 | 0.0030 | 0.0028 | 0.0026 |
|  |  | Pentose phosphate pathway | 0.0072 | 0.0055 | 0.0057 | 0.0073 | 0.0056 | 0.0057 | 0.0071 | 0.0065 | 0.0060 |
|  |  | Propanoate metabolism | 0.0068 | 0.0073 | 0.0067 | 0.0068 | 0.0075 | 0.0067 | 0.0072 | 0.0073 | 0.0067 |
|  |  | Pyruvate metabolism | 0.0098 | 0.0104 | 0.0102 | 0.0097 | 0.0104 | 0.0103 | 0.0097 | 0.0100 | 0.0104 |
|  |  | Starch and sucrose metabolism | 0.0048 | 0.0045 | 0.0049 | 0.0052 | 0.0045 | 0.0050 | 0.0041 | 0.0042 | 0.0054 |
|  | Energy Metabolism | Carbon fixation in photosynthetic organisms | 0.0046 | 0.0051 | 0.0053 | 0.0046 | 0.0050 | 0.0053 | 0.0045 | 0.0047 | 0.0053 |
|  |  | Carbon fixation pathways in prokaryotes | 0.0097 | 0.0095 | 0.0095 | 0.0096 | 0.0095 | 0.0095 | 0.0099 | 0.0097 | 0.0094 |
|  |  | Methane metabolism | 0.0083 | 0.0069 | 0.0067 | 0.0083 | 0.0071 | 0.0068 | 0.0082 | 0.0077 | 0.0070 |
|  |  | Nitrogen metabolism | 0.0086 | 0.0071 | 0.0071 | 0.0085 | 0.0072 | 0.0071 | 0.0086 | 0.0080 | 0.0072 |
|  |  | Oxidative phosphorylation | 0.0104 | 0.0155 | 0.0159 | 0.0103 | 0.0151 | 0.0158 | 0.0109 | 0.0124 | 0.0151 |
|  |  | Photosynthesis | 0.0020 | 0.0027 | 0.0028 | 0.0020 | 0.0027 | 0.0028 | 0.0021 | 0.0023 | 0.0027 |
|  |  | Photosynthesis - antenna proteins | 0.0000 | 0.0000 | 0.0000 | 0.0000 | 0.0000 | 0.0000 | 0.0000 | 0.0000 | 0.0000 |
|  |  | Photosynthesis proteins | 0.0023 | 0.0031 | 0.0032 | 0.0022 | 0.0030 | 0.0032 | 0.0024 | 0.0026 | 0.0031 |
|  |  | Sulfur metabolism | 0.0031 | 0.0041 | 0.0042 | 0.0031 | 0.0040 | 0.0042 | 0.0031 | 0.0035 | 0.0041 |
|  | Enzyme Families | Cytochrome P450 | 0.0000 | 0.0000 | 0.0000 | 0.0000 | 0.0000 | 0.0000 | 0.0000 | 0.0000 | 0.0000 |
|  |  | Peptidases | 0.0149 | 0.0131 | 0.0131 | 0.0149 | 0.0132 | 0.0131 | 0.0148 | 0.0142 | 0.0133 |
|  |  | Protein kinases | 0.0058 | 0.0040 | 0.0039 | 0.0058 | 0.0041 | 0.0039 | 0.0058 | 0.0052 | 0.0040 |
|  | Glycan Biosynthesis and Metabolism | Glycosaminoglycan degradation | 0.0002 | 0.0000 | 0.0000 | 0.0002 | 0.0000 | 0.0000 | 0.0003 | 0.0002 | 0.0000 |
|  |  | Glycosphingolipid biosynthesis - ganglio series | 0.0002 | 0.0000 | 0.0000 | 0.0002 | 0.0000 | 0.0000 | 0.0003 | 0.0002 | 0.0000 |
|  |  | Glycosphingolipid biosynthesis - globo series | 0.0003 | 0.0000 | 0.0000 | 0.0003 | 0.0000 | 0.0000 | 0.0003 | 0.0002 | 0.0001 |
|  |  | Glycosyltransferases | 0.0055 | 0.0029 | 0.0028 | 0.0054 | 0.0030 | 0.0029 | 0.0056 | 0.0046 | 0.0030 |
|  |  | Lipopolysaccharide biosynthesis | 0.0062 | 0.0049 | 0.0049 | 0.0060 | 0.0050 | 0.0049 | 0.0063 | 0.0059 | 0.0047 |
|  |  | Lipopolysaccharide biosynthesis proteins | 0.0095 | 0.0054 | 0.0053 | 0.0093 | 0.0057 | 0.0053 | 0.0096 | 0.0082 | 0.0054 |
|  |  | N-Glycan biosynthesis | 0.0000 | 0.0000 | 0.0000 | 0.0000 | 0.0000 | 0.0000 | 0.0000 | 0.0000 | 0.0000 |
|  |  | Other glycan degradation | 0.0003 | 0.0000 | 0.0000 | 0.0003 | 0.0000 | 0.0000 | 0.0003 | 0.0002 | 0.0001 |
|  |  | Peptidoglycan biosynthesis | 0.0061 | 0.0066 | 0.0067 | 0.0060 | 0.0065 | 0.0067 | 0.0062 | 0.0063 | 0.0066 |
|  |  | Various types of N-glycan biosynthesis | 0.0000 | 0.0000 | 0.0000 | 0.0000 | 0.0000 | 0.0000 | 0.0000 | 0.0000 | 0.0000 |
|  | Lipid Metabolism | alpha-Linolenic acid metabolism | 0.0007 | 0.0007 | 0.0007 | 0.0007 | 0.0007 | 0.0007 | 0.0008 | 0.0007 | 0.0007 |
|  |  | Arachidonic acid metabolism | 0.0005 | 0.0011 | 0.0011 | 0.0005 | 0.0010 | 0.0010 | 0.0005 | 0.0007 | 0.0010 |
|  |  | Biosynthesis of unsaturated fatty acids | 0.0033 | 0.0029 | 0.0028 | 0.0032 | 0.0030 | 0.0028 | 0.0034 | 0.0032 | 0.0028 |
|  |  | Ether lipid metabolism | 0.0000 | 0.0003 | 0.0004 | 0.0000 | 0.0003 | 0.0003 | 0.0000 | 0.0001 | 0.0003 |
|  |  | Fatty acid biosynthesis | 0.0043 | 0.0065 | 0.0067 | 0.0043 | 0.0063 | 0.0067 | 0.0045 | 0.0051 | 0.0065 |
|  |  | Fatty acid metabolism | 0.0053 | 0.0056 | 0.0049 | 0.0053 | 0.0058 | 0.0049 | 0.0056 | 0.0057 | 0.0048 |
|  |  | Glycerolipid metabolism | 0.0026 | 0.0028 | 0.0028 | 0.0026 | 0.0028 | 0.0028 | 0.0025 | 0.0026 | 0.0030 |
|  |  | Glycerophospholipid metabolism | 0.0065 | 0.0056 | 0.0056 | 0.0064 | 0.0056 | 0.0056 | 0.0066 | 0.0063 | 0.0056 |
|  |  | Linoleic acid metabolism | 0.0000 | 0.0004 | 0.0004 | 0.0000 | 0.0003 | 0.0003 | 0.0001 | 0.0001 | 0.0003 |
|  |  | Lipid biosynthesis proteins | 0.0056 | 0.0087 | 0.0088 | 0.0056 | 0.0085 | 0.0088 | 0.0058 | 0.0068 | 0.0084 |
|  |  | Primary bile acid biosynthesis | 0.0002 | 0.0000 | 0.0000 | 0.0002 | 0.0001 | 0.0000 | 0.0003 | 0.0002 | 0.0000 |
|  |  | Secondary bile acid biosynthesis | 0.0002 | 0.0000 | 0.0000 | 0.0002 | 0.0000 | 0.0000 | 0.0003 | 0.0002 | 0.0000 |
|  |  | Sphingolipid metabolism | 0.0001 | 0.0000 | 0.0000 | 0.0001 | 0.0000 | 0.0000 | 0.0001 | 0.0000 | 0.0001 |
|  |  | Steroid biosynthesis | 0.0000 | 0.0000 | 0.0000 | 0.0000 | 0.0000 | 0.0000 | 0.0000 | 0.0000 | 0.0000 |
|  |  | Steroid hormone biosynthesis | 0.0000 | 0.0000 | 0.0000 | 0.0000 | 0.0000 | 0.0000 | 0.0000 | 0.0000 | 0.0000 |
|  |  | Synthesis and degradation of ketone bodies | 0.0001 | 0.0015 | 0.0014 | 0.0001 | 0.0014 | 0.0014 | 0.0001 | 0.0006 | 0.0013 |
|  | Metabolism of Cofactors and Vitamins | Biotin metabolism | 0.0015 | 0.0017 | 0.0018 | 0.0015 | 0.0017 | 0.0018 | 0.0016 | 0.0016 | 0.0017 |
|  |  | Folate biosynthesis | 0.0041 | 0.0052 | 0.0053 | 0.0040 | 0.0050 | 0.0053 | 0.0042 | 0.0045 | 0.0051 |
|  |  | Lipoic acid metabolism | 0.0008 | 0.0007 | 0.0007 | 0.0007 | 0.0007 | 0.0007 | 0.0008 | 0.0007 | 0.0007 |
|  |  | Nicotinate and nicotinamide metabolism | 0.0056 | 0.0042 | 0.0042 | 0.0055 | 0.0043 | 0.0042 | 0.0059 | 0.0053 | 0.0042 |
|  |  | One carbon pool by folate | 0.0039 | 0.0051 | 0.0053 | 0.0038 | 0.0050 | 0.0053 | 0.0039 | 0.0043 | 0.0051 |
|  |  | Pantothenate and CoA biosynthesis | 0.0052 | 0.0057 | 0.0056 | 0.0051 | 0.0057 | 0.0056 | 0.0052 | 0.0054 | 0.0056 |
|  |  | Porphyrin and chlorophyll metabolism | 0.0058 | 0.0068 | 0.0071 | 0.0057 | 0.0067 | 0.0070 | 0.0057 | 0.0060 | 0.0069 |
|  |  | Retinol metabolism | 0.0003 | 0.0005 | 0.0004 | 0.0004 | 0.0005 | 0.0004 | 0.0003 | 0.0004 | 0.0004 |
|  |  | Riboflavin metabolism | 0.0029 | 0.0022 | 0.0021 | 0.0028 | 0.0022 | 0.0021 | 0.0029 | 0.0026 | 0.0022 |
|  |  | Thiamine metabolism | 0.0036 | 0.0034 | 0.0035 | 0.0036 | 0.0034 | 0.0035 | 0.0036 | 0.0035 | 0.0036 |
|  |  | Ubiquinone and other terpenoid-quinone biosynthesis | 0.0051 | 0.0039 | 0.0039 | 0.0050 | 0.0040 | 0.0039 | 0.0051 | 0.0047 | 0.0039 |
|  |  | Vitamin B6 metabolism | 0.0023 | 0.0017 | 0.0018 | 0.0023 | 0.0018 | 0.0018 | 0.0023 | 0.0021 | 0.0018 |
|  | Metabolism of Other Amino Acids | beta-Alanine metabolism | 0.0036 | 0.0042 | 0.0039 | 0.0036 | 0.0043 | 0.0039 | 0.0037 | 0.0040 | 0.0038 |
|  |  | Cyanoamino acid metabolism | 0.0015 | 0.0024 | 0.0025 | 0.0016 | 0.0024 | 0.0025 | 0.0014 | 0.0017 | 0.0025 |
|  |  | D-Alanine metabolism | 0.0012 | 0.0010 | 0.0011 | 0.0012 | 0.0010 | 0.0011 | 0.0013 | 0.0012 | 0.0011 |
|  |  | D-Arginine and D-ornithine metabolism | 0.0000 | 0.0003 | 0.0004 | 0.0000 | 0.0003 | 0.0003 | 0.0000 | 0.0001 | 0.0003 |
|  |  | D-Glutamine and D-glutamate metabolism | 0.0010 | 0.0011 | 0.0011 | 0.0010 | 0.0011 | 0.0011 | 0.0011 | 0.0010 | 0.0011 |
|  |  | Glutathione metabolism | 0.0052 | 0.0056 | 0.0056 | 0.0052 | 0.0056 | 0.0056 | 0.0054 | 0.0055 | 0.0054 |
|  |  | Phosphonate and phosphinate metabolism | 0.0008 | 0.0007 | 0.0007 | 0.0008 | 0.0007 | 0.0007 | 0.0008 | 0.0008 | 0.0007 |
|  |  | Selenocompound metabolism | 0.0038 | 0.0038 | 0.0039 | 0.0038 | 0.0038 | 0.0039 | 0.0039 | 0.0038 | 0.0039 |
|  |  | Taurine and hypotaurine metabolism | 0.0010 | 0.0011 | 0.0011 | 0.0010 | 0.0011 | 0.0011 | 0.0011 | 0.0011 | 0.0011 |
|  | Metabolism of Terpenoids and Polyketides | Biosynthesis of ansamycins | 0.0004 | 0.0004 | 0.0004 | 0.0005 | 0.0004 | 0.0004 | 0.0003 | 0.0003 | 0.0004 |
|  |  | Biosynthesis of siderophore group nonribosomal peptides | 0.0008 | 0.0004 | 0.0004 | 0.0009 | 0.0004 | 0.0004 | 0.0005 | 0.0005 | 0.0005 |
|  |  | Biosynthesis of type II polyketide backbone | 0.0000 | 0.0000 | 0.0000 | 0.0000 | 0.0000 | 0.0000 | 0.0000 | 0.0000 | 0.0000 |
|  |  | Biosynthesis of type II polyketide products | 0.0000 | 0.0000 | 0.0000 | 0.0000 | 0.0000 | 0.0000 | 0.0000 | 0.0000 | 0.0000 |
|  |  | Biosynthesis of vancomycin group antibiotics | 0.0003 | 0.0004 | 0.0004 | 0.0002 | 0.0004 | 0.0004 | 0.0003 | 0.0003 | 0.0003 |
|  |  | Carotenoid biosynthesis | 0.0000 | 0.0003 | 0.0004 | 0.0000 | 0.0003 | 0.0003 | 0.0000 | 0.0001 | 0.0003 |
|  |  | Geraniol degradation | 0.0027 | 0.0031 | 0.0028 | 0.0027 | 0.0032 | 0.0028 | 0.0029 | 0.0030 | 0.0027 |
|  |  | Limonene and pinene degradation | 0.0024 | 0.0029 | 0.0025 | 0.0024 | 0.0030 | 0.0025 | 0.0027 | 0.0028 | 0.0024 |
|  |  | Polyketide sugar unit biosynthesis | 0.0007 | 0.0014 | 0.0014 | 0.0007 | 0.0013 | 0.0014 | 0.0008 | 0.0010 | 0.0013 |
|  |  | Prenyltransferases | 0.0023 | 0.0027 | 0.0028 | 0.0022 | 0.0027 | 0.0028 | 0.0024 | 0.0025 | 0.0028 |
|  |  | Terpenoid backbone biosynthesis | 0.0031 | 0.0045 | 0.0046 | 0.0031 | 0.0044 | 0.0046 | 0.0032 | 0.0036 | 0.0045 |
|  |  | Tetracycline biosynthesis | 0.0015 | 0.0017 | 0.0018 | 0.0014 | 0.0017 | 0.0018 | 0.0016 | 0.0016 | 0.0017 |
|  |  | Zeatin biosynthesis | 0.0003 | 0.0003 | 0.0004 | 0.0002 | 0.0003 | 0.0004 | 0.0003 | 0.0003 | 0.0003 |
|  | Nucleotide Metabolism | Purine metabolism | 0.0200 | 0.0200 | 0.0205 | 0.0197 | 0.0198 | 0.0205 | 0.0205 | 0.0201 | 0.0203 |
|  |  | Pyrimidine metabolism | 0.0136 | 0.0134 | 0.0138 | 0.0134 | 0.0133 | 0.0138 | 0.0140 | 0.0137 | 0.0137 |
|  | Xenobiotics Biodegradation and Metabolism | 1,1,1-Trichloro-2,2-bis(4-chlorophenyl)ethane (DDT) degradation | 0.0000 | 0.0000 | 0.0000 | 0.0000 | 0.0000 | 0.0000 | 0.0000 | 0.0000 | 0.0000 |
|  |  | Aminobenzoate degradation | 0.0020 | 0.0029 | 0.0025 | 0.0020 | 0.0030 | 0.0025 | 0.0021 | 0.0025 | 0.0024 |
|  |  | Atrazine degradation | 0.0001 | 0.0004 | 0.0004 | 0.0001 | 0.0004 | 0.0003 | 0.0000 | 0.0002 | 0.0004 |
|  |  | Benzoate degradation | 0.0025 | 0.0040 | 0.0032 | 0.0026 | 0.0041 | 0.0032 | 0.0025 | 0.0032 | 0.0032 |
|  |  | Bisphenol degradation | 0.0002 | 0.0005 | 0.0004 | 0.0002 | 0.0005 | 0.0004 | 0.0003 | 0.0004 | 0.0003 |
|  |  | Caprolactam degradation | 0.0022 | 0.0017 | 0.0014 | 0.0022 | 0.0019 | 0.0014 | 0.0024 | 0.0022 | 0.0014 |
|  |  | Chloroalkane and chloroalkene degradation | 0.0011 | 0.0019 | 0.0018 | 0.0012 | 0.0019 | 0.0018 | 0.0012 | 0.0014 | 0.0018 |
|  |  | Chlorocyclohexane and chlorobenzene degradation | 0.0001 | 0.0008 | 0.0007 | 0.0001 | 0.0008 | 0.0007 | 0.0001 | 0.0004 | 0.0007 |
|  |  | Dioxin degradation | 0.0006 | 0.0004 | 0.0004 | 0.0007 | 0.0004 | 0.0004 | 0.0005 | 0.0005 | 0.0005 |
|  |  | Drug metabolism - cytochrome P450 | 0.0013 | 0.0019 | 0.0018 | 0.0013 | 0.0019 | 0.0018 | 0.0014 | 0.0016 | 0.0017 |
|  |  | Drug metabolism - other enzymes | 0.0023 | 0.0011 | 0.0011 | 0.0023 | 0.0012 | 0.0011 | 0.0023 | 0.0018 | 0.0012 |
|  |  | Ethylbenzene degradation | 0.0005 | 0.0008 | 0.0007 | 0.0005 | 0.0008 | 0.0007 | 0.0005 | 0.0006 | 0.0007 |
|  |  | Fluorobenzoate degradation | 0.0001 | 0.0004 | 0.0004 | 0.0001 | 0.0004 | 0.0004 | 0.0000 | 0.0002 | 0.0003 |
|  |  | Metabolism of xenobiotics by cytochrome P450 | 0.0013 | 0.0019 | 0.0018 | 0.0013 | 0.0019 | 0.0018 | 0.0013 | 0.0016 | 0.0017 |
|  |  | Naphthalene degradation | 0.0009 | 0.0016 | 0.0014 | 0.0009 | 0.0016 | 0.0014 | 0.0009 | 0.0012 | 0.0014 |
|  |  | Nitrotoluene degradation | 0.0010 | 0.0004 | 0.0004 | 0.0010 | 0.0005 | 0.0004 | 0.0010 | 0.0008 | 0.0004 |
|  |  | Polycyclic aromatic hydrocarbon degradation | 0.0003 | 0.0005 | 0.0004 | 0.0003 | 0.0005 | 0.0004 | 0.0003 | 0.0004 | 0.0004 |
|  |  | Styrene degradation | 0.0003 | 0.0008 | 0.0007 | 0.0003 | 0.0008 | 0.0007 | 0.0003 | 0.0005 | 0.0007 |
|  |  | Toluene degradation | 0.0021 | 0.0018 | 0.0018 | 0.0021 | 0.0019 | 0.0018 | 0.0021 | 0.0020 | 0.0018 |
|  |  | Xylene degradation | 0.0006 | 0.0000 | 0.0000 | 0.0006 | 0.0001 | 0.0000 | 0.0005 | 0.0004 | 0.0001 |

Equations

Equation 1. Residual solids digested by larvae

$$m_{r}=(M-m_{\mathrm{box}}-m_{\mathrm{larvae}})\times(1-MC)$$

Equation 2. Consumption of diet

$$Consumption of diet=\frac{M_{0}-m_{r}}{M_{0}}\times100\%$$

Equation 3. Expected concentration of oxytetracycline in the diet at any time

$$C_{t}=\frac{C_{d}\times m_{r}}{M_{0}}$$

Equation 4. Degradation efficiency and residual oxytetracycline

$$Degradation efficiency=\frac{C_{0}-C_{t}}{C_{0}}\times100\%$$

$$Residual oxytetracycline=100\%-Degradation efficiency=\frac{C_{t}}{C_{0}}\times100\%$$

where $C_{0}$ is the initial concentration of oxytetracycline, $M$ is the total weight, $m_{box}$ is the weight of the breeding box, $m_{larvae}$ is the weight of the larvae, $MC$ is the moisture content of the diet, $M_{0}$ is the total initial dry weight, and $C_{d}$ is the determined concentration of oxytetracycline.

# Figures


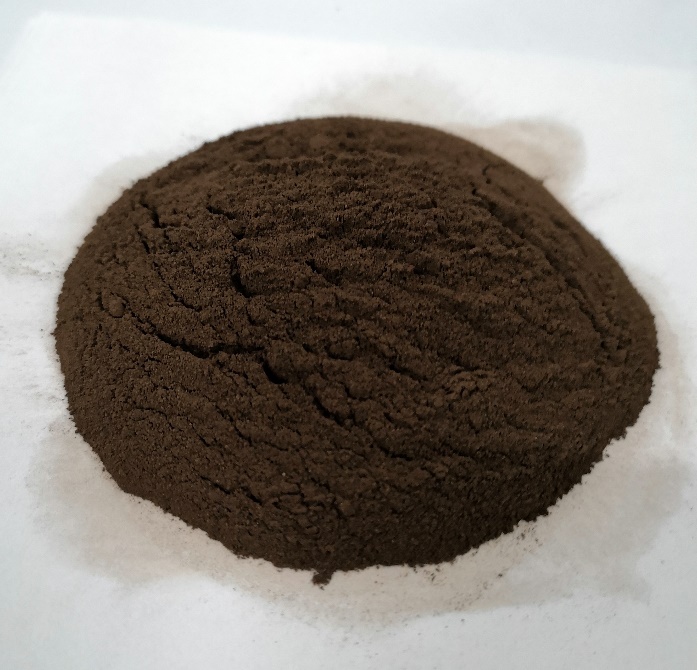


Figure 1. The diagram of dried oxytetracycline bacterial residue.

(a) (b)

Figure 2. Changes in (a) the consumption of substrate and oxytetracycline bacterial residue and (b) the fresh weight of larvae. Each point is the average concentration in triplicate samples (mean ± SE). “**” and “***” indicate *P* < 0.01 and *P* < 0.001, respectively. **” indicate *P* < 0.05, *P* < 0.01 and P < 0.001, respectively. The soya, OBRlow and OBRhigh represented treatments containing only soybean meal, low amount of OBR and high amount of OBR, respectively.

Figure 3. Rarefaction curves of all the samples (label: 0.03). soya, OBRlow and OBRhigh represented the treatments containing only soybean meal, low amount of OBR and high amount of OBR, respectively; _1, _3, _6 represented the first day, third day and sixth day, respectively; 1-, -2, -3 represented the repetition of the experiment.


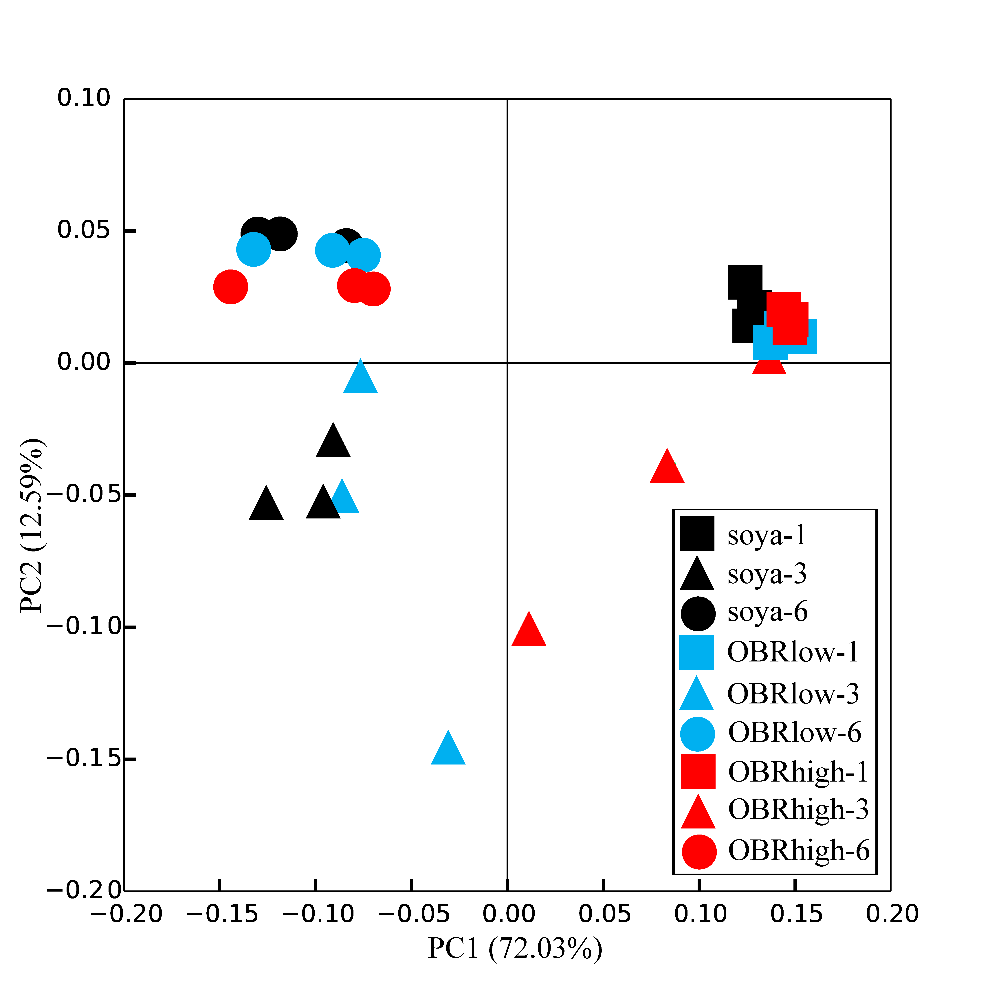


Figure 4. Principal coordinate analysis (PCoA) of larval gut samples using the weighted UniFrac distance (Adonis test, *P*＜0.001). The soya, OBRlow and OBRhigh represented treatments containing only soybean meal, low amount of OBR and high amount of OBR, respectively; 1, 3, 6 represented the first day, third day and sixth day, respectively.

Figure 5. The gut microbiota at the phylum level (mean, n = 3). The soya, OBRlow and OBRhigh represented treatments containing only soybean meal, low amount of OBR and high amount of OBR, respectively; 1, 3, 6 represented the first day, third day and sixth day, respectively.

Figure 6. Heat maps showing the enrichment of taxa (mean, n = 3). The soya, OBRlow and OBRhigh represented treatments containing only soybean meal, low amount of OBR and high amount of OBR, respectively; 1, 3, 6 represented the first day, third day and sixth day, respectively.

Figure 7. (a) Proportions of total antibiotic resistance genes (ARGs) detected in the gut, classified by the resistance mechanism. (b) Proportions of total ARGs detected in the gut, classified by the resistance to antibiotics. An ARG was considered detected when it was amplified in one sample; because many resistance genes were targeted by multiple primers, the detection of the same gene by multiple primer sets was counted as the detection of only a single unique resistance gene (mean, n=3).

Figure 8. Abundance of ARGs and MGEs in the guts of larvae. Absolute copy numbers of antibiotic resistance genes (ARGs) and mobile genetic elements (MGEs) (mean ± SE, n=3). “**” indicates *P* < 0.01. The soya, OBRlow and OBRhigh represented treatments containing only soybean meal, low amount of OBR and high amount of OBR, respectively; 1, 3, 6 represented the first day, third day and sixth day, respectively.

Figure 9. Normalized copy numbers of tetracycline resistance gene occupancy (mean ± SE, n=3). “***” indicates P < 0.001. Different letters (T groups) indicate significant differences (*P* < 0.05). The soya, OBRlow and OBRhigh represented treatments containing only soybean meal, low amount of OBR and high amount of OBR, respectively; 1, 3, 6 represented the first day, third day and sixth day, respectively.

Figure 10. The profiles of MGEs and ARGs that confer resistance to each class of antibiotics. Each column is labeled with the sample name, each row is the result from a single primer set, and the values plotted are normalized gene copy numbers (copies per cell). All primer sets (153 detected ARGs and 10 detected MGEs) that showed amplification in at least one sample are shown (mean, n=3). The soya, OBRlow and OBRhigh represented treatments containing only soybean meal, low amount of OBR and high amount of OBR, respectively; 1, 3, 6 represented the first day, third day and sixth day, respectively.

Figure 11. Gene profiles of ARGs and MGEs detected in the guts of larvae. Each column is labeled with the sample name, each row is the result from a single primer set, and the values plotted are normalized gene copy numbers (copies per cell). All primer sets (153 detected ARGs and 10 detected MGEs) that showed amplification in at least one sample are shown (mean, n=3). The soya, OBRlow and OBRhigh represented treatments containing only soybean meal, low amount of OBR and high amount of OBR, respectively; 1, 3, 6 represented the first day, third day and sixth day, respectively.

Figure 12. Correlation of the normalized gene copy numbers of ARGs with the normalized copy numbers of transposases or of the class 1 integron-integrase gene. Unweighted, the data used were logarithmically transformed. *P* < 0.001 indicates a significant correlation.
